# Risk Factors and Treatment of Chylothorax After Minimally Invasive Esophagectomy for Esophageal Cancer

**DOI:** 10.7759/cureus.65606

**Published:** 2024-07-28

**Authors:** Yuma Tsuchitani, Yohei Ozawa, Yusuke Taniyama, Hiroshi Okamoto, Chiaki Sato, Hirotaka Ishida, Takashi Kamei

**Affiliations:** 1 Department of Surgery, Tohoku University Graduate School of Medicine, Sendai, JPN; 2 Department of Surgery, Tohoku University Hospital, Sendai, JPN

**Keywords:** postoperative complicaiton, chylothorax, minimally invasive esophagectomy (mie), esophagectomy, esophageal neoplasms

## Abstract

Background

Postoperative chylothorax is a rare but life-threatening complication of esophagectomy. However, due to its rarity, researching the risk factors and selecting appropriate treatment options has been limited.

Methods

This study included 727 patients with esophageal cancer who underwent minimally invasive esophagectomy at our hospital. To detect the risk factors for chylothorax, we divided the patients into two groups, with and without postoperative chylothorax. We then compared patient characteristics, tumor-specific variables, and operative details. Subsequently, we analyzed the peri-treatment characteristics and outcomes for the three distinct treatment options we had chosen: surgery, conversion (the group that finally underwent surgery after unsuccessful conservative treatment), and conservative.

Results

Of the 727 patients, 18 (2.5%) developed a chylothorax. The mean BMI was lower (20.3 vs. 21.9, p=0.057), and more cases of thoracic duct resection were found in the chylothorax group (33.3% vs. 6.2%, p=0.001), with statistical significance. Multivariate analysis identified thoracic duct resection as a risk factor (adjusted odds ratio, 6.83). The drainage volume two days after chylothorax was higher in the surgery group, although the difference was not statistically significant (surgery group, 1,405 ml vs. conversion group, 260 ml vs. conservative group, 310 ml; p=0.073). The surgery group had the shortest median postoperative hospital days among these groups (21.5 as compared to 102 and 25.0 days in the conversion and conservative groups, respectively; p<0.001). None of the patients died during their hospital stays.

Conclusion

Thoracic duct resection during the initial minimally invasive esophagectomy was an independent risk factor for chylothorax. If drainage volume does not decrease on the second day, early surgery may lead to earlier discharge.

## Introduction

Chylothorax after esophagectomy is a relatively rare and life-threatening complication; it is often difficult to treat, and the treatment could be prolonged [1,2]. The incidence of postoperative chylothorax after esophagectomy is reportedly approximately 2.0-8.8% [2-12]. Once a chylothorax occurs, the patient readily suffers from hypovolemia, malnutrition, and immunosuppression, which can further lead to postoperative secondary complications [13,14]. Furthermore, low BMI [8,11], squamous cell carcinoma [6,7], neoadjuvant therapy [7,10], high intraoperative fluid balance [10], and thoracic duct resection [10] have been reported as risk factors for chylothorax. However, these have not been adequately investigated because of the rare incidence of chylothorax.

Currently, most surgeries for esophageal cancer involve minimally invasive esophagectomy (MIE), which offers advantages such as a lower risk of pulmonary infections and a shorter hospital stay than conventional thoracotomy [15]. However, to the best of our knowledge, no studies have examined the risk of chylothorax specific to MIE.

Generally, the treatment options for chylothorax can be divided into a conservative approach, based on thoracic drainage, and a surgical approach such as thoracic duct ligation. Since the most common cause of chylothorax is thoracic duct injury caused during surgical manipulation, thoracic duct ligation is considered the most reliable treatment method [16]. However, it is crucial to minimize unnecessary reoperations because patients with esophageal cancer often exhibit characteristics such as advanced age, malnutrition, and respiratory and cardiac problems. Accordingly, appropriate treatment options should be selected for each patient on a case-by-case basis. The present study aimed to identify the risk factors for chylothorax in MIE and evaluate the indications for managing chylothorax, particularly with a specific focus on surgical intervention.

## Materials and methods

This was a retrospective study conducted at Tohoku University Hospital, Sendai, Miyagi, Japan, to examine the risk factors and treatment options for chylothorax after MIE. The study was approved by the Ethics Committee of the Graduate School of Medicine, Tohoku University (approval number: 2019-1-429), and informed consent was obtained from all patients for participation and publication before initial surgery.

Between January 2007 and September 2021, 816 patients with esophageal cancer underwent esophagectomy at Tohoku University Hospital. Of these, 89 patients who underwent open thoracotomy or transhiatal esophagectomy were excluded. Finally, 727 patients who underwent MIE were enrolled. We retrospectively reviewed medical records of patients undergoing MIE, extracted preoperative characteristics, tumor-specific variables, and operative details, and divided the patients into two groups: patients with and without chylothorax, and evaluated risk factors for chylothorax.

Surgical procedure

**Figure 1:**
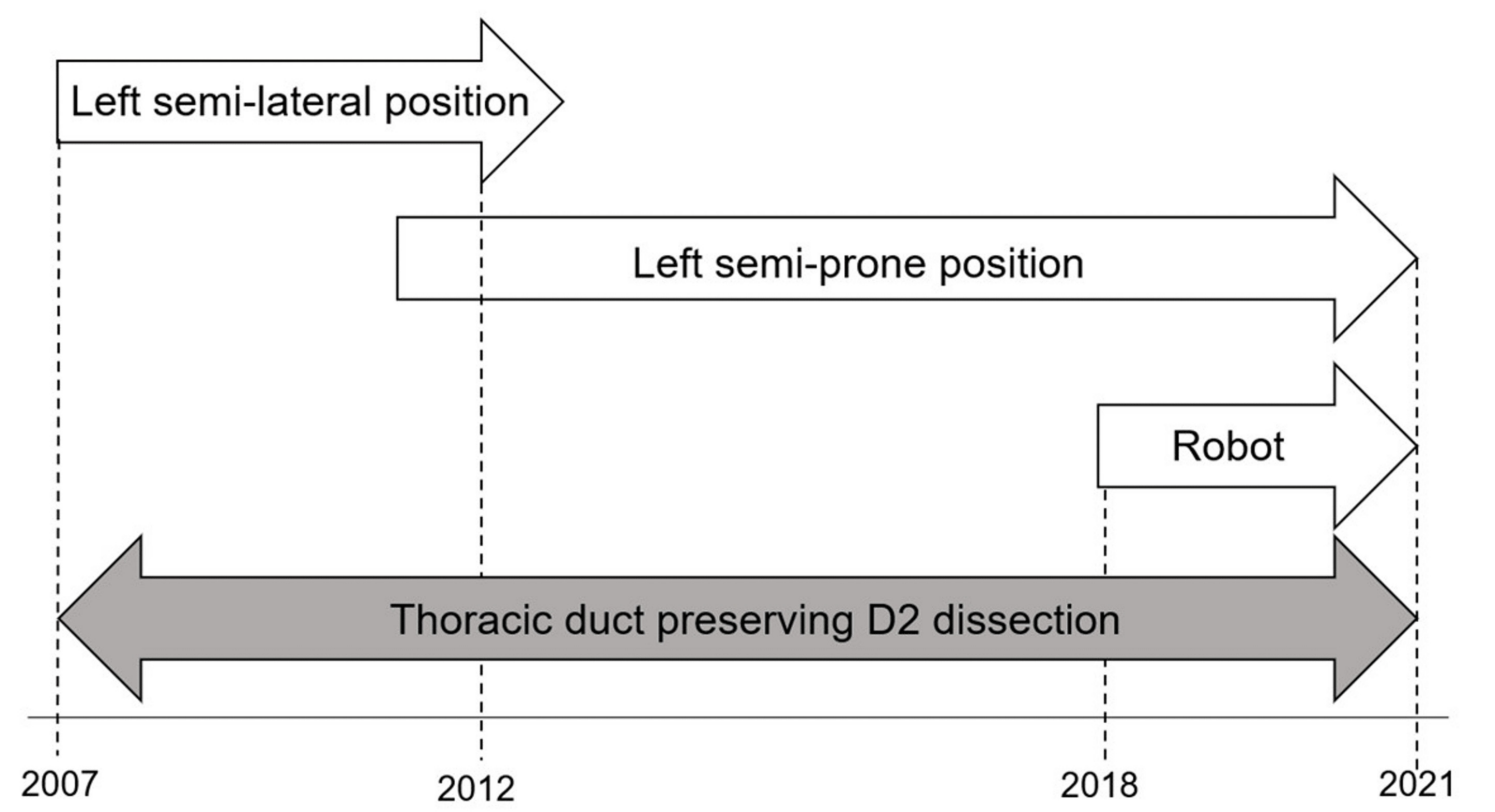
Transition of surgical procedures in Tohoku University Hospital, Sendai, Japan

In our hospital, McKeown esophagectomy was performed routinely in the left semi-lateral position between 2007 and 2012, which has been changed to the left semi-prone position since 2013. In 2018, we introduced robot-assisted esophagectomy as a new surgical approach. Abdominal manipulation was performed using hand-assisted laparoscopic surgery except in patients who concurrently have gastric cancers or a history of laparotomy. We generally performed D2 dissection according to the Japanese Classification of Esophageal Cancer guidelines of that time [17,18]. In principle, we strived to preserve the thoracic duct, except in cases of advanced cancers that are deemed incurable without thoracic duct resection. Gastric tube reconstruction was also routinely performed. Figure 1 shows the transition of MIE in our hospital. The operation for chylothorax was performed with en masse supradiaphragmatic ligature.

Chylothorax diagnosis and assessment of treatment

Chylothorax was diagnosed clinically based on the observable change in the character of chest tube drainage, which typically transformed into a milky-white appearance, along with consideration of the drainage volume. When the character of drainage fluid was not clear milky-white and the diagnosis was troubling, we collected drainage fluid and diagnosed chylothorax according to the following criteria: pleural fluid triglycerides (TG) > 110 mg/dL, ratio of pleural fluid TG to serum TG > 1, or confirmation of chylomicrons in the pleural fluid when pleural fluid TG is 50-110 mg/dL [19].

To assess the treatment of chylothorax, patients who developed chylothorax were classified further into the following three groups according to the treatment method: (i) Surgery group, which comprised patients who underwent reoperation within one week after diagnosis of chylothorax, (ii) Conversion group, which comprised patients who underwent reoperation after unsuccessful conservative treatment, and (iii) Conservative group, which comprised patients treated conservatively and improved chylothorax. Data on the drainage volume for three days from the date of diagnosis of chylothorax, duration of drainage, and postoperative stay were extracted and compared between the different treatment groups. The duration of drainage was defined from initial esophagectomy to drain removal.

Statistical analysis

Continuous variables were represented using means and standard deviations and analyzed using Student’s t-test, or alternatively, represented using medians and ranges or interquartile ranges and analyzed using the Mann-Whitney U test. Categorical variables were represented using frequencies and proportions and analyzed using Fisher’s exact test. The Kruskal-Wallis test was used to compare continuous variables among the three groups. Risk factors for chylothorax were assessed using logistic regression analysis adjusted for age, sex, and previously reported risk factors. All statistical analyses were performed using SAS Studio software (SAS Institute, Cary, North Carolina, United States). All p-values were two-tailed, and statistical significance was set at p < 0.05.

## Results

Patient characteristics

Table 1 shows the preoperative characteristics and tumor-specific variables of the two groups. Of the 727 patients, 18 (2.5%) developed a chylothorax. Although not statistically significant, the patients with chylothorax had a lower BMI (20.3 vs. 21.9; p=0.057). There were no statistically significant differences in terms of tumor factors; however, over 70% of the patients in the chylothorax group presented with stage 3 or more advanced disease compared to 56.7% in the group without chylothorax.

**Table 1:** Preoperative characteristics and tumor-specific variables BMI: body mass index; SCC:squamous cell carcinoma; AC: adenocarcinoma; NAC: neoadjuvant chemotherapy; CRT: chemoradiotherapy; RT*: *radiotherapy; IQR:interquartile range

Variables	Chylothorax (n=18)	No chylothorax (n=709)	p-value
Sex, n (%)			0.76
Male	14 (77.8)	580 (81.9)	
Female	4 (22.2)	129 (18.1)	
Age (mean ± SD)	63.6±8.8	66.2±8.5	0.21
BMI (mean ± SD)	20.3±2.1	21.9±3.5	0.057
Smoking history, n (%)	13 (76.5)	579 (76.5)	0.52
Hypertension, n (%)	7 (38.9)	269 (38.0)	1.00
Diabetes, n (%)	2 (11.1)	91 (12.6)	1.00
Histology, n (%)			0.97
SCC	16 (88.9)	570 (80.9)	
AC	1 (5.6)	71 (10.1)	
Special types	1 (5.6)	63 (9.0)	
Pre-treatment, n (%)			0.78
None	6 (33.3)	253 (37.5)	
NAC	7 (38.9)	277 (41.0)	
CRT or RT	5 (27.8)	145 (21.4)	
Location, n (%)			0.65
Cervical esophagus	0 (0)	7 (1.0)	
Upper thoracic esophagus	3 (16.7)	80 (11.4)	
Middle thoracic esophagus	10 (55.6)	325 (46.4)	
Lower thoracic esophagus	5 (27.8)	231 (33.1)	
Abdominal esophagus	0 (0)	57 (8.2)	
Tumor length (mm), median (IQR)	40.0 (25.0–60.0)	35.0 (23.0–48.0)	0.34
Clinical T-factor			0.49
1-2	6 (33.3)	295 (42.2)	
3-4	12 (66.7)	401 (57.8)	
Clinical N-factor			0.78
Negative	5 (27.8)	216 (30.9)	
Positive	13 (72.2)	483 (69.1)	
Clinical stage			0.23
0– II	5 (27.8)	301 (43.2)	
III–IV	13 (72.2)	397 (56.8)	

Operative details

Table 2 shows the operative details for the two groups. The patients with chylothorax were significantly more likely to undergo thoracic duct resection than those without chylothorax (33.3% vs. 6.2%; p=0.001). No statistically significant differences were observed for other variables. A distinct evaluation of lymph node 112, situated close to the thoracic duct, demonstrated no discernible difference in cases with and without positive metastasis.

**Table 2:** Surgical details for both groups The patients with chylothorax were significantly more likely to undergo thoracic duct resection than those without chylothorax. IQR: interquartile range

Variables	Chylothorax (n=18)	No chylothorax (n=709)	p-value
Thoracic duct resection, n (%)	6 (33.3)	44 (6.2)	<0.001
Number of dissection lymph nodes, median (IQR)	43.0 (24.0–51.0)	38 (27.0–50.0)	0.85
Thoracic operation time (minutes), mean ± SD	279.1±92.0	267.0±74.0	0.51
Total operation time (minutes), mean ± SD	606.4±120.2	613.8±103.4	0.77
Thoracic amount of blood loss (ml), median (IQR)	38.5 (15–175)	37.0 (14–150)	0.87
Total amount of blood loss (ml), median (IQR)	120.0 (47–235)	220.0 (107–417)	0.058
Amount of fluid infusion (ml/kg/h), median (IQR)	7.28(5.51–7.96)	6.50 (5.06–8.24)	0.52
Number of surgeon’s experience > 10 Cases	14 (77.8)	517 (73.9)	1.00
#112 lymph node metastasis	1 (5.56)	29 (4.11)	0.54

Univariate and multivariate analysis of risk factors for chylothorax

Table 3 summarizes the results of univariate and multivariate analyses. Univariate analysis showed statistically significant differences in BMI (OR, 0. 84, p=0.042) and thoracic duct resection (OR = 7.56, p<0.001). After adjusting for age, sex, and risk factors reported previously (neoadjuvant therapy and histology), the thoracic duct resection showed a statistically significant difference between the two groups (Adjusted OR (aOR), 6.83, p<0.001) and was independently associated with chylothorax. However, after adjustment, no significant differences were observed in BMI. Furthermore, no significant differences were observed between squamous cell carcinoma and neoadjuvant therapy.

**Table 3:** Univariate and multivariate analyses, adjusting for age, sex, and risk factors reported previously. In multivariate analysis, the adjusted OR for thoracic duct in the chylothorax group was significantly higher than in the no chylothorax group. SCC: squamous cell carcinoma; AC:adenocarcinoma; NAC: neoadjuvant chemotherapy

Variables	Univariate analysis			Multivariate analysis		
	OR	95％ CI	p-value	Adjusted OR	95％ CI	p-value
BMI	0.84	0.72–0.99	0.042	0.86	0.72–1.02	0.085
Thoracic duct resection	7.56	2.71–21.09	<0.001	6.83	2.40–19.44	<0.001
Histology (SCC vs. AC)	0.50	0.07–3.83	0.51	0.66	0.07–5.87	0.71
NAC	1.07	0.36–3.23	0.91	0.79	0.24–2.53	0.75
Age	-	-	-	0.97	0.92–1.03	0.30
Sex	-	-	-	0.96	0.28–3.26	0.95

Comparison of treatments

Table 4 compares treatments. The surgery group had four patients, the conversion group had three patients, and the conservative treatment group had 11 patients. There was no significant difference in the drainage volume on the first day among the three groups (surgery group, 1,025 ml vs. conversion group, 900 ml vs. conservative group, 940 ml; p=0.86). However, the surgery group had a larger drainage volume on the second (1,405 ml vs. 260 ml (conversion group) vs. 310 ml (conservative group); p=0.073) and third days (850 ml vs. 300 ml (conversion group) vs. 290 ml (conservative group); p=0.096), although the difference was not significant. Postoperative length of stay in the surgery group was the shortest among the three groups (21.5 vs. 102 (conversion group) vs. 25.0 days (conservative group), p<0.001).

**Table 4:** Comparison of chylothorax treatments † From initial esophagectomy to drain removal

Variables	Surgery (n=4)	Conversion (n=3)	Conservative (n=11)	P-value
Thoracic duct at IME, n (%)				1.00
Resection	1 (25.0)	1 (33.3)	4 (36.4)	
Preservation	3 (75.0)	2 (66.7)	7 (63.6)	
Days to diagnosis, median (range)	1.5 (1–2)	2 (2–7)	3.0 (1–13)	0.37
Drainage amount after diagnosis of chylothorax (ml), median (range)				-
Day 1	1025 (760–1880)	900 (540–1950)	940 (240–2700)	0.86
Day 2	1405 (760–1470)	260 (250–670)	310 (90–1950)	0.073
Day 3	850 (550–1320)	300 (40–680)	290 (100–1300)	0.096
Drainage duration^†^, median (range)	7 (6–9)	28 (22–60)	7 (5–17)	<0.001
Postoperative hospital stay, median (range)	21.5 (15–28)	102 (32–105)	25.0 (14–45)	<0.001

## Discussion

In this study, we examined the risk factors of chylothorax following MIE and chylothorax treatment. Previously reported risk factors include squamous cell carcinoma, adjuvant therapy, high intraoperative fluid balance, thoracic duct resection, and low BMI [6-8,10,11]. However, it should be noted that all these studies included patients who underwent open esophagectomy. We found that in MIE, combined resection of the thoracic duct is a risk factor for postoperative chylothorax. This finding is consistent with those of previous studies. Previous studies have included both thoracotomy and MIE, but this study focused on MIE only and, to the best of our knowledge, is the first report of such an observation in patients with MIE.

 At our hospital, we preserve the thoracic duct, unless it is deemed necessary to do otherwise, to cure locally advanced cancers. When the thoracic duct is resected, it is ligated or clipped to prevent lymphatic leakage. In general, postoperative chylothorax after thoracic duct resection is thought to be caused by loose ligatures or clipping. However, the network of small lymphatic vessels surrounding the main trunk of the thoracic duct may sustain damage during dissection, resulting in chylothorax [10]. Even with the magnification afforded by the thoracoscopic approach, it remains challenging to visualize these small lymphatic vessels; therefore, thoracic duct resection may also be a risk factor for MIE.

Although the difference was not statistically significant, a lower BMI was associated with a higher risk of chylothorax, consistent with previous reports [8,11]. A study examining the incidence of postoperative complications of esophageal cancer in four BMI groups showed a large difference in terms of the incidence of chylothorax between BMI <18.5 and BMI >30 at 21.0% and 3.3%, respectively [20]. Among individuals with a higher BMI, the thoracic duct is protected by fatty tissue, possibly reducing the incidence of chylothorax compared with that in underweight patients [8,20]. Therefore, the procedure in the thoracic duct area should be performed using a more protective approach in underweight patients or cases of advanced cancer necessitating thoracic duct resection.

To prevent postoperative chylothorax, surgeons must be familiar with the wide variety of anatomical variations of the thoracic duct. The thoracic duct is the main trunk of the lymphatic system and collects lymph from the lower and upper left body. It begins in the cisterna chyli and runs through the mediastinum, usually opening into the left venous angle [21]. Adachi et al. classified the thoracic duct into nine types and reported anatomical variations in 13% of cases [22]. In our hospital, we have performed preoperative magnetic resonance thoracic ductography (MRTD) and reconstructed the thoracic ducts using three-dimensional images to understand their relationship with the surrounding organs before surgery [23]. MRTD can help identify the anatomy of the thoracic duct. This enabled us to preoperatively identify cases involving duplicate thoracic ducts and cases with an opening into the right venous angle. A study has reported a 14% rate of anatomical variation in the delineation of the thoracic duct using MRTD [24] Another study compared the incidence of postoperative complications with and without MRTD before esophagectomy and found a significantly lower incidence of chylothorax in the MRTD group [25].

Currently, there is a lack of consensus on the management of chylothorax. Clinicians initially attempt conservative treatment; however, if ineffective, surgical treatment is generally performed. Selle et al. recommended surgical intervention under the following conditions: (i) when milky drainage exceeded 1,500 ml and persisted for five days or more, (ii) when milky drainage persisted for two weeks or more, and (iii) patients had worsening nutritional status [26]. Recently, Cerfolio et al. suggested that surgery should be indicated when the average amount of thoracic drainage is >1,000 ml during the first seven days after surgery [16]. In our hospital, there are no established surgical indications for postoperative chylothorax. Therefore, each case was clinically evaluated to determine whether revision surgery should be performed.

In the current study, we tended to choose surgical treatment if the drainage volume did not decrease two or three days after the diagnosis of chylothorax. Favorable outcomes observed in all patients in the surgery group indicate that early surgery could be a viable option for patients, particularly when the amount of drainage from the chest tube does not decrease despite medical treatment such as fasting, total parenteral nutrition, and somatostatin administration. Continuous milky drainage, despite a decrease in the amount of drainage, is a serious concern and prolonged conservative treatment could worsen the patient’s condition. The above reports by Selle et al. [26] and Cerfolio et al. [16] recommended one to two weeks of conservative treatment, but these reports included not only patients with esophageal cancer but also patients with lung cancer and traumatic chylothorax. However, patients with esophageal cancer often have problems with malnutrition and poor cardiopulmonary function, which can easily worsen their general condition. Therefore, in postoperative esophageal cancer, when a patient has an overall favorable condition, it is advisable to opt for surgical treatment before the general condition worsens due to chylothorax. In all cases of postoperative chylothorax, we performed en masse supradiaphragmatic ligature. This method has been shown to be effective even if the leak is not identified preoperatively or intraoperatively [27].

Despite the success demonstrated; this study has some limitations. First, this study’s findings might not be applicable to different demographic groups or healthcare settings, given that it was conducted in a single university hospital in Japan. Second, some missing data existed because this was a long-term retrospective study. We excluded and analyzed each variable on a per-variable basis. Third, because chylothorax is a rare complication, this study might have been underpowered to detect certain differences or associations. Thus, a larger number of cases are required.

## Conclusions

Thoracic duct resection during MIE can significantly increase the risk of developing postoperative chylothorax, and early surgical intervention for chylothorax in cases without decreased drainage may lead to a better outcome if the patient is not in poor general condition. Through these contributions, our study not only advances the current understanding of postoperative complications in esophageal cancer surgery but also serves as a crucial steppingstone for future research and clinical practice enhancements in this field.
